# Facile Synthesis of Surface-Modified Carbon Quantum Dots (CQDs) for Biosensing and Bioimaging

**DOI:** 10.3390/ma13153313

**Published:** 2020-07-25

**Authors:** Łukasz Janus, Julia Radwan-Pragłowska, Marek Piątkowski, Dariusz Bogdał

**Affiliations:** Faculty of Chemical Engineering and Technology, Cracow University of Technology, 31-155 Krakow, Poland; j.radwan@doktorant.pk.edu.pl (J.R.-P.); marek.piatkowski@pk.edu.pl (M.P.); pcbogdal@cyf-kr.edu.pl (D.B.)

**Keywords:** nanoparticles for imaging and sensing, bioinspired materials, materials for active optical elements

## Abstract

Recently, fluorescent probes became one of the most efficient tools for biosensing and bioimaging. Special attention is focused on carbon quantum dots (CQDs), which are characterized by the water solubility and lack of cytotoxicity. Moreover, they exhibit higher photostability comparing to traditional organic dyes. Currently, there is a great need for the novel, luminescent nanomaterials with tunable properties enabling fast and effective analysis of the biological samples. In this article, we propose a new, ecofriendly bottom-up synthesis approach for intelligent, surface-modified nanodots preparation using bioproducts as a raw material. Obtained nanomaterials were characterized over their morphology, chemical structure and switchable luminescence. Their possible use as a nanodevice for medicine was investigated. Finally, the products were confirmed to be non-toxic to fibroblasts and capable of cell imaging.

## 1. Introduction

The most advanced biosensing and bioimaging methods rely on the nanotechnology solutions [1,2,3]. Nanomaterials are widely applied for detecting assays production with active optical elements. The most promising ones involve the application of quantum dots (QDs) due to their unique properties such as superior resistance to photobleaching [4,5,6,7]. Tools for biodetection based on QDs enable reversible quantification and detection of various biomolecules attracts a lot of scientists’ attention since they have a lot of advantages comparing to traditionally used classic organic fluorophores. Quantum dots exhibit specificity to certain biologically relevant substances and have reasonably high quantum yield and broad absorption spectra [8]. On the contrary to conventional dyes, these nanomaterials are characterized with tunable, size-dependent, narrow emission spectra and long fluorescence lifetime [9,10]. The optoelectronic properties of the QDs come from the transformation in size-dependent energy levels systematic transformations, which are correlated with the size of quantum dots [8]. Such a phenomenon is called the quantum confinement effect [8,9,10,11,12,13,14,15]. Quantum dots may be applied in bioimaging and biodiagnostics [16,17,18,19]. Various types of these nanomaterials were successfully used for this purpose such as CdS, CdSe, CdTe, MoS_2_ or PbS. Typical size of their inorganic core ranges from 4 to 10 nm. However, semiconductive QDs application may be limited by low solubility in aqueous solutions, poor chemical stability and cytotoxicity [20,21,22]. Importantly, under in vivo conditions they may undergo structural changes and contribute to reactive oxygen species generation [20]. Thus, for their safe use they must be covered with biocompatible coatings or shells. Noteworthy, such modifications significantly increase their size even up to 30 nm, which negatively affects specificity to certain biomolecules. Nanodots may penetrate cell membrane via the endocytosis process, however it is strictly dependable on the nanoparticle size and shape as well as chemical composition [23,24]. For example, carboxylated quantum dots may enter the cell due to the clathrin-mediated endocytosis. Thus, any surface modification may lead to the non-desired cell-nanoparticle interaction [23,24]. Therefore, preparation of the QDs with an inorganic core with the size below 10 nm and the satisfactory level of biocompatibility maintain a great challenge for the scientists. Additionally, their susceptibility to fluorophores emission fluctuations called photoblinking is a major drawback of these materials in comparison with other types of nanodots [20,21,22].

Carbon quantum dots (CQDs) are a specific group of the previously described nanomaterials that are spherical objects of the size below 10 nm with a carbon core. CQD’s optical properties depend on their surface, thus their modification with various biomolecules enables precise tailoring of their sensing behavior. They may have a crystalline or amorphous structure. Carbon quantum dots fluorescence can be explained by surface defects or conjugated π-bonds bandgap transitions [20,25,26,27,28,29,30]. The nanodots can be prepared via two different approaches, namely top down and bottom up. In most cases, they undergo post-modification [27,28]. First attempts of CQDs preparation involved chemical oxidation and electrochemical reactions. Other popular approaches include laser ablation and arch discharge. They enable fast obtainment of the nano-objects with mostly a crystalline structure and uniform size. However, these methods that are commonly called top-down, require second step enabling semiproducts functionalization due to the unsatisfactory level of quantum yield [27,28,29]. Therefore, new pathways have been developed. Bottom-up methods enable simultaneous carbon core formation and surface modification. As a raw material organic matter rich in carbon atoms may be applied.

CQDs can be obtained via the solvothermal route, in the field of microwave radiation, thermal decomposition or by the template approach. Aforementioned ways of nanodots preparation are considered as ecofriendly, since they enable the use of waste biomass of various origin as CQDs precursors such as coffee grounds, fruit and vegetable peels and others. Very often also polysaccharides, peptides, antibodies or amino acids (cysteine) are also applied due to the presence of heteroatoms such as oxygen, nitrogen or sulphur, which play a crucial role in the enhancement of the carbon dots fluorescence properties [29,30,31,32]. The CQD’s surface is rich in various functional groups such as amino, hydroxyl or carboxyl, which are responsible for their hydrophilicity, which is a significant advantage over graphene quantum dots (GQDs), which are known to be poorly soluble in water. They are also crucial to proceed surface passivation. Nanodots can be doped by heteroatoms or grafted with various compounds to increase quantum yield or affinity to certain biomolecules, which make them an ideal candidate for the most advanced and sensitive tools for biosensing and bioimaging [30,31,32,33,34,35]. Additionally, they exhibit excellent stability in aqueous media.

CQDs due to their excellent photoluminescence are used for various cells’ visualization [35,36,37]. Affinity to the biological structures can be increased trough NH_2_ groups grafting using amines such as 2-ethylenediamine, poly(ethyleneamine) or trimethylamine. Incorporation of nitrogen atoms improves also resistance to photobleaching and imagining resolution. Nanodots are useful also in the optical imaging of cancer cells and biomarkers. High availability of the raw material combined with an easily accessible apparatus required for their preparation such as household microwaves makes carbon quantum dots a very cheap material with outstanding characteristics comparing to traditional organic fluorophores used in biology and medicine. Carbon quantum dots exhibit satisfactory brightness, imaging sensitivity and photostability, which are crucial for effective bioimaging. Additionally, they are resistant to the biodegradation caused by various enzymes. Importantly, CQDs compared to commercially available QDs do not bioaccumulate in tissue and enable even very small biological structures visualization using confocal microscopy. Depending on the CQDs type, they may visualize nuclei or cytoplasm of the human cells, fungi and bacteria. They do not change cells morphology. What is important, low cytotoxicity of the carbon quantum dots is not correlated with the raw material used for their obtainment [35,36,37].

Currently, quantum dots are successfully applied in the production of biosensors. They can be used in the cellular labeling, nucleic acid detection, immunoassays and diagnostic assays. Such systems are mostly based on the in solid/liquid interface interactions. The highest development was achieved in the case of optical fibers and microbeads. Assays based on QD’s solutions are highly problematic due to the necessity of the colloid stability maintenance. On the other hand, assays of the solid-phase type require high quantities of quantum dots for immobilization. Another type of biosensing device relies on the microfluidic systems [20,21,22].

Carbon quantum dots have a great potential in the field of biology and medicine due to their numerous advantages. Nevertheless, there are still many issues to be solved to increase their applicability in biomedical applications such as biosensing and bioimaging [20,29].

The aim of the following research was to obtain a novel type of carbon quantum dots with switchable fluorescence according to the Green Chemistry principles applicable in diagnostics and medicine. The CQDs were obtained as a result of the facile reaction by the hydrothermal method using chitosan, glucosamine, cellulose and glucose as raw materials and urea, urotropin modifying agents. The products were characterized over their chemical structure, size and morphology. Moreover, their photoluminescence behavior was investigated. CQDs potential in biodetection using model protein (albumin), glucose and vitamin C as well as chromium (VI) ions was verified. The lack of cytotoxicity was confirmed using L929 cells by the XTT ((2,3-Bis-(2-Methoxy-4-Nitro-5-Sulfophenyl)-2H-Tetrazolium-5-Carboxanilide)) assay.

## 2. Materials and Methods

### 2.1. Materials

Glucose, acetic acid, sodium hydroxide (NaOH), citric acid, ammonia, hydrochloric acid (HCl), potassium dichromate and ascorbic acid were delivered by POCH, Gliwice, Poland. Glucosamine, chitosan, cellulose, XTT assay and PBS (phosphate buffer solution) was delivered by Sigma-Aldrich, Poznań, Poland. Dulbecco’s modified Eagle medium (DMEM), streptomycin/penicillin and fetal bovine serum was delivered by Polgen, Łódź, Poland. Dialysis tubing (molecular weight cut off (MWCO) 500–1000 Da) was purchased from VWR, Gdańsk, Poland.

### 2.2. Methods

#### 2.2.1. CQDs Synthesis

Carbon quantum dots were synthesized in a stainless-steel hydrothermal reactor (50 mL volume, 30 bar) (Toption, Shanxi, China) with a Teflon reaction vessel. To the starting material in the amount of 0.5 g (glucose, glucosamine, cellulose and chitosan) 0.5 mL of the hydrochloric acid and 0.1 g of the modifying agent (urea, urotropin) (Sigma-Aldrich, Poznań, Poland) was added. The reactors were placed in oven at 180 °C for 12 h. After the carbonization process suspensions were sonificated using Emmi-20 HC ultrasound bath (EMAG Polska, Juszczyn, Poland) and filtered by using membrane filters (VWR, Gdańsk, Poland) with a 0.22 µm pores diameter. All samples were neutralized by using 5% NaOH solution by using an Elmetron CX-551 pH meter (Elmetron, Zabrze, Poland) and the solutions were dialyzed by using dialysis tubing (MWCO 500–1000 Da) and water (VWR, Gdańsk, Poland) as the purifying agent for 4 days to remove small molecular weight compounds and inorganic ions. Prepared CQDs solutions were diluted for spectroscopic analysis. The reaction parameters and samples composition are given in Table 1.

#### 2.2.2. Spectroscopic and Chemical Structure Study

Absorption spectra were obtained by using UV-Vis Agilent 8453 diode-array spectrophotometer (Santa Clara, CA, USA). Chemical structure study was made by using Thermo Nicolet Nexus-470 FTIR (Fourier-transform infrared spectroscopy) spectrophotometer (Thermo Fisher Scientific, Waltham, MA, USA) with a diamond ATR adapter. Fluorescence characteristic, fluorescence quantum yield and pH-dependence fluorescence study at pH = 4, 5, 6, 7, 8 and 9 were made by FP-750 Jasco Spectrofluorometer (JASCO, Tokio, Japan). For the fluorescence studies, the CQDs solutions were diluted so their absorbance was A = 0.05. During pH-dependence studies the samples were at 365 nm. Buffer solutions were prepared by using the CX-551 Elmetron pH-meter. The fluorescence quantum yield of CQDs was measured by using a comparative method with quinine sulfate (Sigma-Aldrich, Poznań, Poland) in 0.1 M sulfuric acid (Sigma-Aldrich, Poznań, Poland) as a standard (fluorescence quantum yield (QY) = 0.54) by the method described in the previous article [28]. The QY was calculated using the following Equation (1):

(1) $${\mathrm{QY}}_{s}=Q_{r}\left(\frac{A_{r}}{A_{s}}\right)\left(\frac{E_{s}}{E_{r}}\right){\left(\frac{\eta_{s}}{\eta_{r}}\right)}^{2}$$

QY = Fluorescence quantum yield;

η = Refractive index of the solvent;

A = Absorbance of the solution;

E = Integrated fluorescence intensity of the emitted light.

Subscripts ‘r’ and ‘s’ refer to the quinine sulfate (reference) and evaluated sample respectively.

#### 2.2.3. CQDs TEM Morphology Analysis

For CQDs morphology examination the JEOL JEM2100 HT CRYO LaB6 Transmission Electron Microscope (Jeol USA, lnc., Peabody, MA, USA) was used. Small drops of solution of CQDs were transferred on measurement holders and left for water evaporation.

#### 2.2.4. Biodetection Study

Albumin, vitamin C, potassium dichromate and glucose solutions were prepared by dissolving reagents in PBS buffer (pH = 7.4). The CQDs and analyzed samples were mixed together and the fluorescence intensity was measured by using the JASCO FP-750 spectrofluorometer (JASCO, Tokio, Japan). The detection limit was determined by preparing solutions of decreasing concentration. For the given concentration values, the dependence of the fluorescence intensity on the analyte concentration was rectilinear. The excitation wavelength was λ = 365 nm, whereas the emission wavelength was λ = 480 nm.

#### 2.2.5. Cytotoxicity Study

The CQD’s cytotoxicity study was conducted on L929 mouse fibroblasts by using an XTT (Sigma-Aldrich, Poznań, Poland) assay. The cell line was purchased from Sigma-Aldrich, Poznań, Poland, which distribute this cell line from European Collection of Authenticated Cell Cultures (ECACC), a part of Public Health England for research use only. The cell line number and origin are ECACC 85011425, respectively mouse C3H/An connective tissue. 

For this study cell proliferation was measured for 48 h. For the experiments, various concentrations were used (0.05–0.30 mg/mL). All CQDs for investigation in the cytotoxicity study were sterilized by using sterile membrane filters with a 0.22 µm pore diameter.

#### 2.2.6. Cell Visualization by Fluorescence Microscopy

Cell luminescence observations were made by a Delta Optical inverted microscope (Planeta Oczu, Zielona Góra, Poland) with an epifluorescence adapter (400× magnification). For this study cells were incubated in a 24 multi-well plate (Nest, GenoPlast Biochemicals Tomasz Schröder, Rokocin, Poland) for 24 h with 0.2 mg/mL CQDs solutions. After that time CQDs/SBF solution was removed and labeled cells were washed 2 times with a PBS buffer. For cell luminescence observations 460/520 nm filters were used.

## 3. Results and Discussion

**Figure 1:**
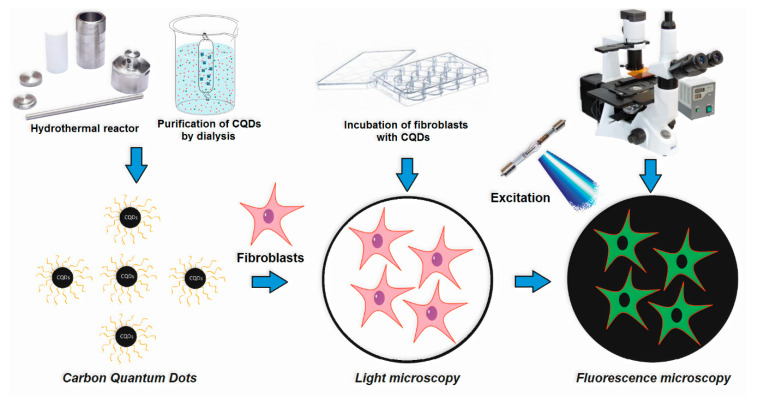
General strategy for the carbon quantum dot’s (CQD’s) preparation and application.

**Figure 2:**
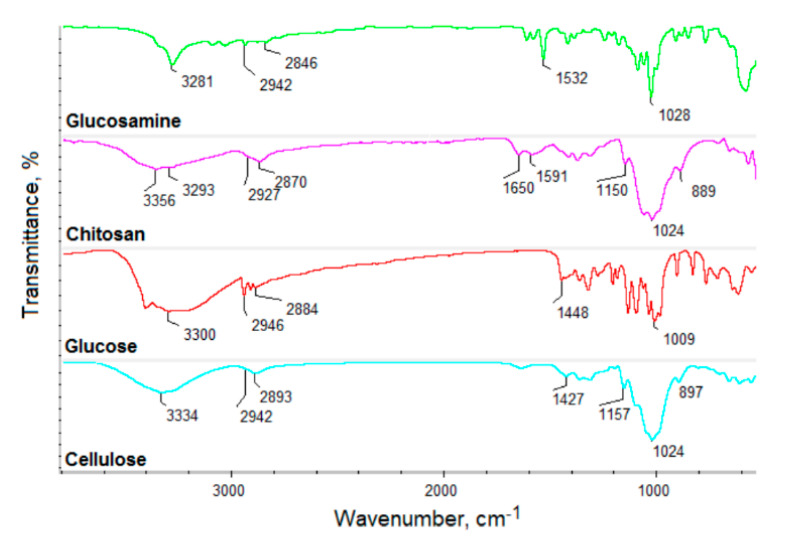
FT-IR spectra of the starting materials for the CQD’s preparation.

**Figure 3:**
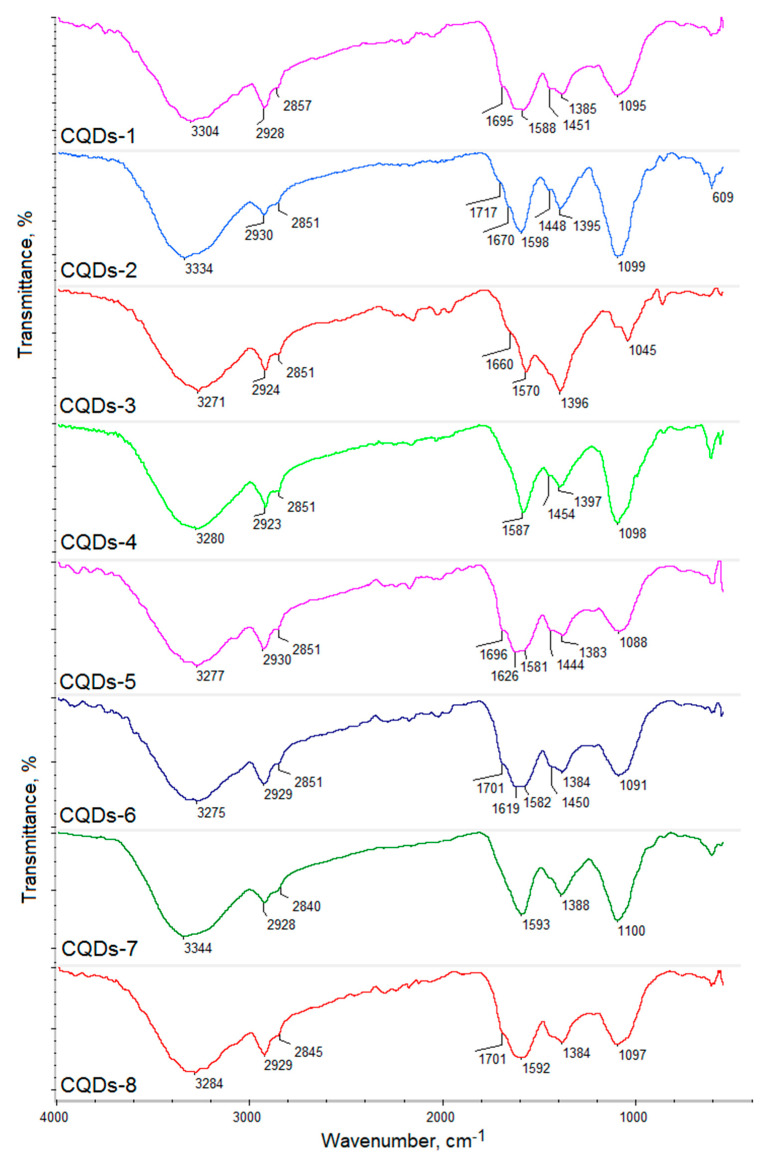
FT-IR spectra of the prepared samples.

Figure 1 presents the general CQD’s preparation pathway and potential application. Figure 2 shows the FT-IR spectra of the raw materials used for CQD’s preparation, namely glucosamine, chitosan, glucose and cellulose. Figure 3 presents the FT-IR spectra of the prepared CQDs. One may observe that the spectra of the carbon nanodots significantly differed from the raw materials. All samples exhibited some typical bands coming from hydrophilic functional groups such as amino, carboxyl and hydroxyl, which are typical for carbon quantum dots and are responsible for their water solubility and interactions with cell membranes. Importantly, the peaks intensity is much higher after reaction, which proves heteroatoms CQDs doping. However, their intensity varied in different products, which is caused by the type of the raw material as well as the modifying agent that were used for their obtainment. Samples CQDs-1 and CQDs-2 were prepared using glucosamine as a precursor, while CQDs-3–CQDs-4 samples were synthesized using chitosan, which is a polymer containing glucosamine and *N*-acetylaminoglucose mers. Their FT-IR spectra show bands coming from hydroxyl and carboxyl groups (CQDs-1—3304 cm^−1^; CQDs-2—3334 cm^−1^; CQDs-3—3271 cm^−1^ and CQDs-4—3280 cm^−1^, respectively) and bands coming from free amino groups at 1588 cm^−1^ (CQDs-1), 1598 cm^−1^ (CQDs-2), 1570 cm^−1^ (CQDs-3) and 1587 cm^−1^ (CQDs-4), respectively. Importantly, for the samples prepared from glucose and cellulose, they also contained nitrogen atoms due to the successful modification with urea and urotropin, which confirmed bands at 1581 cm^−1^ (CQDs-5), 1582 cm^−1^ (CQDs-6), 1593 cm^−1^ (CQDs-7) and 1592 cm^−1^ (CQDs-8). The spectra also show bands typical for amide bonds at 1695 cm^−1^ (CQDs-1), 1670 cm^−1^ (CQDs-2), 1660 cm^−1^ (CQDs-3), 1626 cm^−1^ (CQDs-5) and 1619 cm^−1^ (CQDs-6). All samples also exhibited bands coming from -CH_2_- and CH_3_ aliphatic groups in the range between 2930 and 2840 cm^−1^. The chemical structure is typical for the CQDs and corresponds to other researchers’ data [20,25]. The presence of various functional groups is important for the selective interactions with various molecules. Importantly, the presence of the free amino groups is desirable for cell labeling and cell imaging applications.

**Figure 4:**
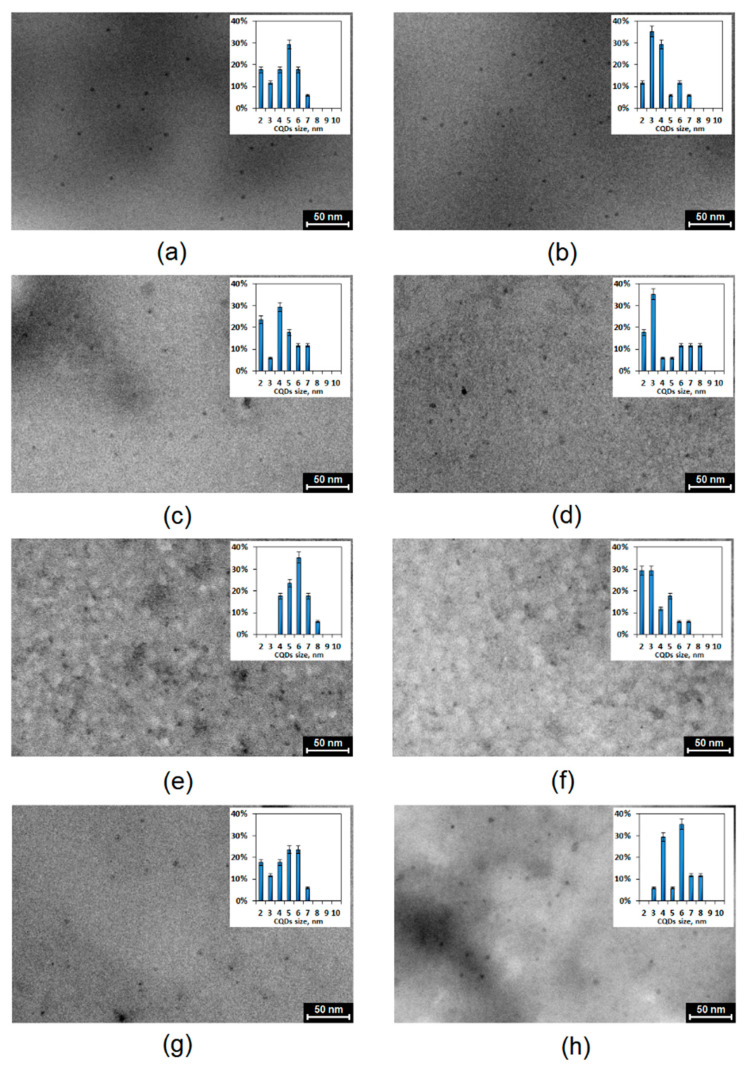
TEM images of the prepared samples (**a**)—CQDs-1; (**b**)—CQDs-2; (**c**)—CQDs-3; (**d**)—CQDs-4; (**e**)—CQDs-5; (**f**)—CQDs-6; (**g**)—CQDs-7 and (**h**)—CQDs-8.

The unique luminescence phenomena were observed for the dots of the size below 10 nm and were size-dependent. Figure 4 presents TEM images of the nanoproducts. It revealed that all of the prepared samples had a round shape and diameter typical for CQDs. In general, the formation of the CQDs occurred due to the thermal degradation and carbonization of the raw material resulting in the formation of carbon nanogranules. The samples prepared from aminoglucose (CQDs-1 and CQDs-2) were uniform in size and shape, which was around 6 nm. It may be noticed that the choice of the modifying agent somehow affected the process of the CQDs formation. The nanodots prepared from the polymer (chitosan) were characterized by lower homogeneity in terms of their morphology. Their size ranged between 4 and 8 nm. The samples obtained using glucose (CQDs-5, CQDs-6) also had a round shape and dimensions below 10 nm. Additionally, in this case a correlation between the type of modifying agent used and dot’s size can be observed, since again the use of urotropin resulted in the preparation of the smaller particles. The dots based on the cellulose exhibited a spherical shape, size below 10 nm and had quite small uniformity. The impact of the modifier on the CQDs size is not observed in the case of CQDs obtained from the polymer. Such results prove that both the concentrations and synthesis parameters such as time and temperature were chosen properly since the conditions were suitable for carbon core formation due to the nucleation [20,23,27,28].

**Figure 5:**
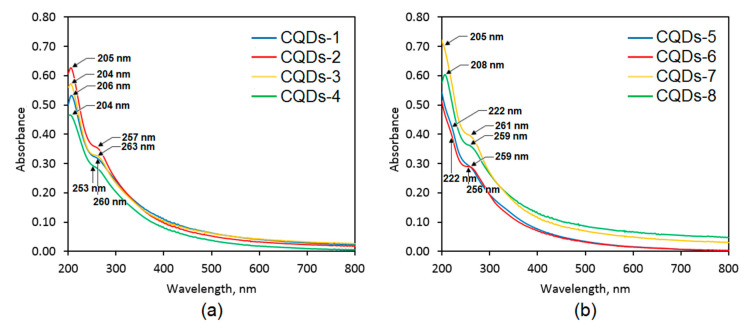
UV-Vis spectra of the prepared samples: (**a**)—CQDs-1–CQDs-4 and (**b**)—CQDs-5–CQDs-8.

Figure 5 presents the UV-Vis spectra of the prepared nanomaterials. All of the CQDs were characterized by two peaks visible approximately at 205 nm and 260 nm, which are typical for the electron transitions occurring in carbons with sp^2^ hybridization (π–π* type) present in the carbon core of the nanodots. The UV-Vis spectra are characteristic for carbon quantum dots [20,25,30]. The highest intensity is observed in the case of the CQDs-2 and CQDs-7 samples. Importantly, for all of the samples except CQDs-5 and CQDs-6 (glucose-derived) there was a noticeable impact of the modifying agent used. However, it was not uniform and depended on the raw material chosen for the carbonization. For the samples obtained from the polymers, namely chitosan and cellulose one may notice that modification with urea positively affected optical properties of the nanodots. On the contrary, in the case of the CQDs prepared from aminoglucose (low molecular weight substance) better optical properties are observed for the product obtained using urotropin as a modifier than urea. The results correspond to other researchers’ data [23].

**Figure 6:**
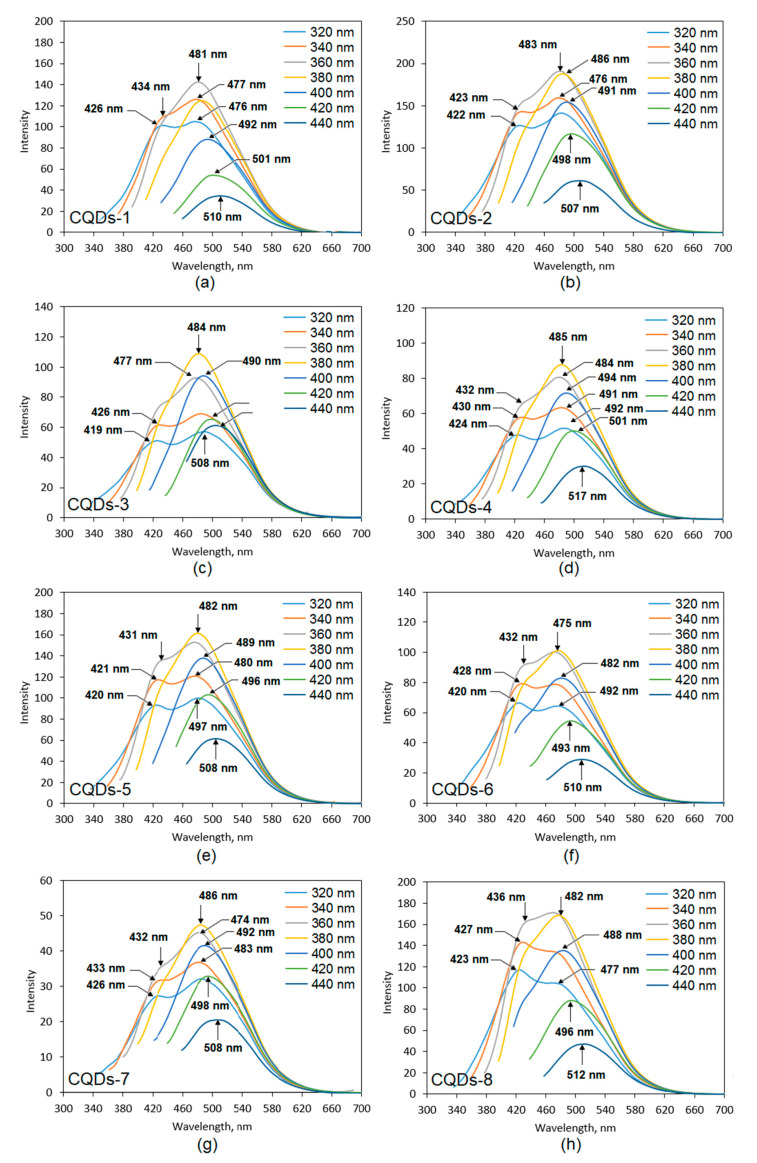
Fluorescence spectra of the prepared samples: (**a**)—CQDs-1; (**b**)—CQDs-2; (**c**)—CQDs-3; (**d**)—CQDs-4; (**e**)—CQDs-5; (**f**)—CQDs-6; (**g**)—CQDs-7 and (**h**)—CQDs-8.

**Figure 7:**
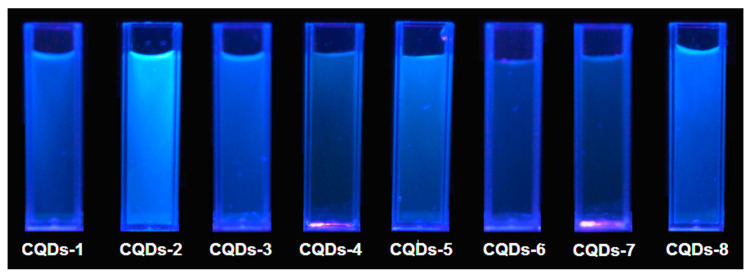
Prepared CQDs irradiated with the diode (365 nm).

One of the crucial properties for the CQDs application is their fluorescence. Figure 6 presents fluorescence spectra collected using various excitation wavelengths (from 320 to 440 nm) of the prepared samples. It can be observed that fluorescence intensity depends on the wavelength at which the CQDs were excited, which is typical for this type of nanomaterials. One may observe that the highest PL (photoluminescence) intensity was obtained at different wavelengths for various samples. In the most cases, the nest results were collected for excitation at 360 nm and 380 nm. The lowest PL intensity was observed for the excitation at 440 nm except for the CQDs-3 sample (320 nm). All of the samples exhibited two maxima when excited at 320 nm, 340 nm and 360 nm, except for CQDs-1 sample, which had two maxima when excited at 320 nm and 340 nm. The obtained results were typical for carbon quantum dots and could be attributed both to the various functional groups present on the surface of the CQDs as well as due to the quantum effect, which is assigned to the different sizes of the particles [5,6,9]. The spectra given in the Figure 6 shows that the highest fluorescence intensity was obtained for the CQDs-2 sample, which was prepared from aminoglucose and modified with urotropin and CQDs-8 prepared from cellulose and modified with the same compound. The CQDs prepared from aminoglucose but modified with urea (CQDs-1) also exhibited high PL intensity. The lowest PL was noticed for the CQDs-7 sample. The fluorescence of other samples was on a satisfactory level. What is interesting, the choice of the modifying agent affected the PL properties in various ways and there was no general tendency for all of the samples, similarly as in the case of the optical characteristics of the products. The fluorescence of the prepared samples was a result of the disruption of the crystalline structure along with the band gap transitions (conjugated π-type bonds), which is characteristic for carbon quantum dots [5,6,14,20,25,29,30]. Figure 7 presents the CQDs solutions irradiated with the commercially available diode, which exhibit blue fluorescence at λ = 365 nm.

**Figure 8:**
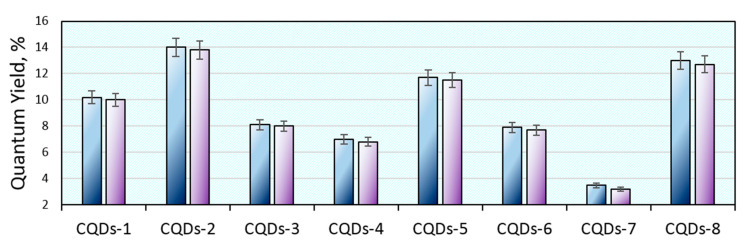
Quantum yield of the prepared samples and its stability after 7 days.

Figure 8 shows the results of the quantum yield study of the freshly prepared CQDs and after 7 days. It can be noticed that QY values corresponded to the PL intensity given in Figure 6. The highest quantum yield was obtained for the CQDs-2 (14%) and CQDs-8 (13%) samples. The lowest QY is observed in the case of sample CQDs-7 (only 3.5%). Such values are typical for carbon quantum dots prepared from the waste biomass and well-purified from unreacted or semi-carbonized residues, which the presence often overstates the QY thus giving misleading results. Importantly, the CQDs that are dedicated for various industrial applications such as photocatalysis or ion detection very often do not undergo intense purification by long-term dialysis so to shorten their preparation procedure. However, the biomedical applications require high purity and a well-defined chemical composition. The investigated CQDs exhibited excellent QY stability over time (up to 0.3% after one week), which is a great advantage comparing to the traditional organic dyes that easily undergo photobleaching. Thus, they have great potential in biomedical applications [6,9,14,29,30,32].

**Figure 9:**
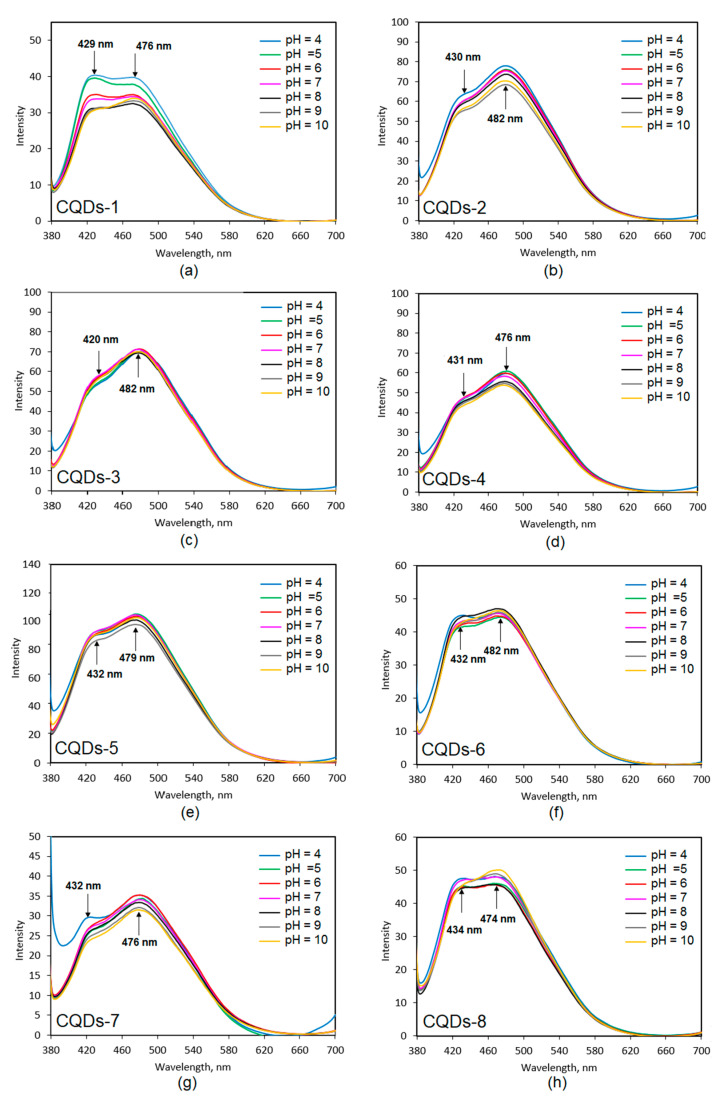
Fluorescence dependence on pH of the prepared samples: (**a**)—CQDs-1; (**b**)—CQDs-2; (**c**)—CQDs-3; (**d**)—CQDs-4; (**e**)—CQDs-5; (**f**)—CQDs-6; (**g**)—CQDs-7 and (**h**)—CQDs-8.

Carbon quantum dots prepared by top down methods without post modification in most cases do not contain functional groups that are responsible for pH-sensitivity. The fluorescence quenching in some applications is a highly desired feature since it enables the collection of valuable data by non-invasive methods without sample destruction. Figure 9 presents results of the CQDs PL dependence on pH. All samples exhibited two peak maxima at approximately 430 nm and 480 nm, which are independent from the pH of the medium. It can be noticed that the behavior in the solutions of various acidity differed depending on the sample type and was assigned to the type of both the raw material and modifying agent used. The pH-sensitivity is observable in the case of samples 1, 2 and 8, which could be attributed to the high amount of free carboxyl, amino and hydroxyl groups. In the case of other nanodots their PL intensity change was very low or negligible. One may observe that the PL intensity was the highest under acidic conditions, which can be explained by the protonation of the NH_2_ groups, which leads to the increase of the charge. The higher charge was responsible for a stronger repulsion of the CQDs leading to higher PL intensity. The results corresponded to the FT-IR spectra (Figure 3) since there was a correlation between pH dependence and peaks intensity (NH_2_, OH and COOH groups). The only exception was sample 8 where the highest intensity was observed for the pH = 10. Most of the human body fluids were characterized by pH around 7. Therefore, the PL intensity change observed due to the pH increase or decrease can be helpful during biodetection, bioimaging or biochemical processes studies especially these on poisoning, cancer or diabetes since it may provide data on the biological material condition rapidly. The highest pH-sensitivity is observed for the CQDs-2 sample, which is also characterized by the highest quantum yield and fluorescence intensity [29,31].

**Figure 10:**
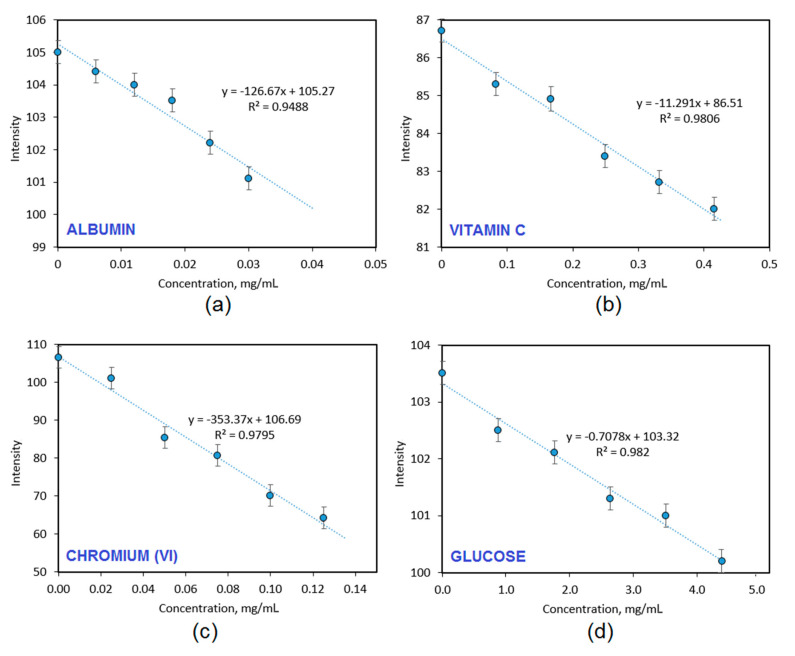
The study on biodetection abilities carried out in PBS of the CQDs-2 sample: (**a**)—detection of the albumin; (**b**)—detection of the vitamin C; (**c**)—detection of the chromium (VI) ions and (**d**)—detection of the glucose.

The prepared carbon quantum dots due to their surface modification exhibited interesting fluorescence properties, which could be assigned to the presence of the various functional groups. Figure 10 presents results of the biodetection capability studies of the CQDs-2 sample, which had the highest fluorescence quantum yield and exhibited pH-dependent PL. It can be noticed that the nanodots have different sensitivity to various biologically relevant molecules and ions. The CQDs have a great ability to detect model protein-albumin (0.01 mg/mL minimal detection level) and chromium (VI) ions. The fluorescence quenching occurred due to the interactions between free amino groups from the surface of carbon quantum dots, which attracted negatively charged carboxyl groups present in the protein structure leading to the loss of radiation absorption ability. Excellent detection was also possible in the case of chromium ions (0.02 mg/mL minimal detection level), which could be also explained by the chelating properties caused by the presence of functional groups present on the CQDs surface, which can coordinate Cr^6+^ ions and act as an organic ligand. The CQDs-2 sample exhibited a moderate ability of vitamin C biodetection (0.1 mg/mL minimal detection level) whereas the PL intensity decreased when the glucose was quite low. The results given in Figure 10 confirm the potential of the prepared CQDs in their application in diagnostics and biodetection since selective fluorescence quenching is observed, which is their reaction to the substance presence in the phosphate buffer solution. Importantly, the biodetection is able to perform in the biological matrix [22,27,28,30,34].

**Figure 11:**
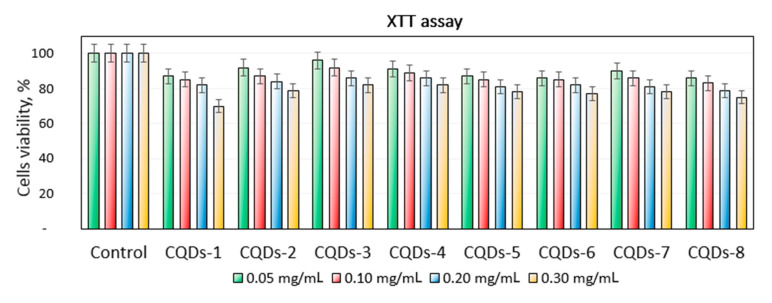
XTT assay carried out on mouse fibroblasts (L929 cell line).

Figure 11 revealed the study on the prepared CQDs cytotoxicity to L929 mouse fibroblasts. It can be noticed that all of the samples could be considered as non-toxic since the amount of metabolically active cells was above 70% in each case. However, the correlation between their concentration and toxicity can be observed. Importantly, CQDs in the concentration that can be considered as biosafe were 0.30 mg/mL. The highest change in the number of viable cells was spotted for the CQDs-1 sample. Such results confirm the right choice of the raw materials. The highest number of the viable cells was obtained for the samples prepared from chitosan and glucose as raw materials. There was no unequivocal correlation between the type of modifying agent and cytotoxicity. The results are typical for the carbon nanodots and correspond to other researchers’ data and confirm their potential in biomedical applications [29,30].

**Figure 12:**
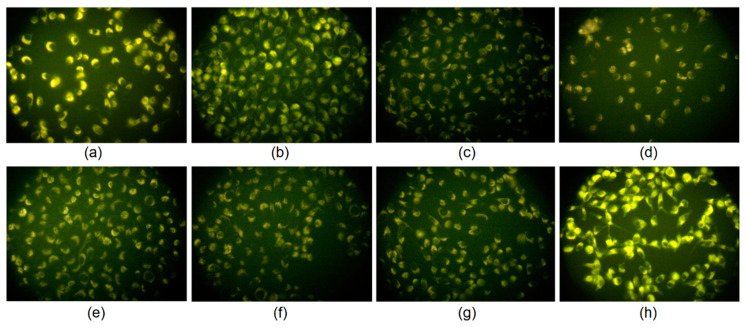
Mouse fibroblasts visualized using prepared CQDs: (**a**)—CQDs-1; (**b**)—CQDs-2; (**c**)—CQDs-3; (**d**)—CQDs-4; (**e**)—CQDs-5; (**f**)—CQDs-6; (**g**)—CQDs-7 and (**h**)—CQDs-8.

The prepared CQDs are dedicated for biomedical applications. Due to their unique properties such as tunable fluorescence and a size below 10 nm they can be applied in cell labeling. Figure 12 shows the results of the bioimaging of skin fibroblasts. It can be observed that all types of the CQDs were capable of cell membrane penetration, which can be assigned to the appropriate size and the presence of the positively charged NH_2_ groups, which are attracted electrostatically by negatively charged fibroblasts surface components. It can be observed that the CQDs emitted fluorescence under standard conditions using a commercially available inverted microscope, which proves their applicability in various biomedical applications. Interestingly, one may observe that the nanodots did not penetrate the nuclear envelope. The CQDs stained the cytoplasm. The microphotographs suggest that the carbon nanoparticles might interact selectively with vimentin, which is a structural protein forming cytoskeleton. The efficiency of the staining was mostly correlated with the quantum yield of the samples. The best results were obtained for the CQDs-8 sample, which can be caused by the highest ability to penetrate the cell membrane by this type of carbon quantum dots due to the most appropriate morphology and chemical structure. One may assume that too high of an amount of the free amino groups may result in too strong electrostatic interactions of the CQDs and cell membrane components thus hampering their penetration inside the cell. The most likely, carbon quantum dots entered cells due to the clathrin-mediated endocytosis or macropinocytosis pathway, which is the most typical for this type of nanomaterial and was confirmed to occur for the QDs of the size 8 nm [23,24]. The results show that the CQDs enable vital staining of the eukaryotic cells and may constitute an interesting alternative to the commercially available products such as expensive antibodies [35,36,37,38].

## 4. Conclusions

The aim of this work was to prepare biocompatible CQDs by the facile method using waste biomass as a raw material. The obtained nanodots were characterized by tunable fluorescence and quantum yield up to 14%. The nanomaterial exhibited pH-sensitivity and the ability of biologically relevant substances detection such as proteins. Moreover, all samples were non-cytotoxic to L929 mouse fibroblasts. The potential in biomedical applications was confirmed by investigating CQD’s ability to penetrate cell membranes and emit fluorescence thus staining cytoplasm. The obtained results show that proposed carbon nanodots can be applied in medicine and pharmacy as elements of advanced sensors, optical fibers or for cell bioimaging and biochemical processes study.

## Figures and Tables

**Table 1:** Samples description.

Sample	C Source	Modifying Agent	Carbonizing Acid	Time (h)	Temperature (°C)
CQDs-1	Glucosamine	Urea	HCl	12	180
CQDs-2	Glucosamine	Urotropin	HCl	12	180
CQDs-3	Chitosan	Urea	HCl	12	180
CQDs-4	Chitosan	Urotropin	HCl	12	180
CQDs-5	Glucose	Urea	HCl	12	180
CQDs-6	Glucose	Urotropin	HCl	12	180
CQDs-7	Cellulose	Urea	HCl	12	180
CQDs-8	Cellulose	Urotropin	HCl	12	180
